# Supplementary material for: Validation Study of Italian Version of Inventory for Déjà Vu Experiences Assessment (I-IDEA): A Screening Tool to Detect Déjà Vu Phenomenon in Italian Healthy Individuals
Source: Behav Sci (Basel). 2017 Aug 6;7(3):50. doi: 10.3390/bs7030050 (PMC5618058; doi:10.3390/bs7030050)
Supplement: Supplementary file 1 [file behavsci-07-00050-s001.pdf]

# Questionario per la valutazione del *Deja-vu* (I-IDEA)

- **Nome:**

- **Cognome:**

- **Data di nascita:**

- **Sesso:**

- ☐ M
- ☐ F

**Scolarità:**

Continua……..

## A
## INTRODUZIONE

Il presente questionario riguarda una sensazione che crediamo sia familiare a molti. Quasi ciascuno di noi ha avuto, almeno una volta nella vita, la sensazione di aver già vissuto precedentemente un evento, un pensiero o una sensazione esattamente nello stesso modo, sebbene in realtà si trattasse della prima volta. E' come se riconoscessimo qualcosa, anche se sappiamo che è impossibile.

Questa sensazione di "riconoscimento" è chiamata "déjà vu"
"**Déjà vu"** letteralmente significa "già visto".
**Per ogni risposta, è molto importante la tua prima impressione. Non è necessario pensare a lungo! Assicurati di leggere l'introduzione prima di ciascuna serie di domande.**
**Per favore, non saltare nessuna domanda!**
Per rispondere a ciascuna domanda, cerchia la risposta corrispondente.
**Per esempio: Se desideri rispondere "Si": o Si**

**1. Ti è mai sembrato di aver già vissuto una sensazione o situazione esattamente allo stesso modo, sebbene in realtà si trattasse della prima volta?**
**(Nota: se non sei sicuro, per favore rispondi "Mai")**
**o** Mai
**o** Si, molto raramente (meno di 1 volta l' anno)
**o** Si, qualche volta (poche volte l'anno)
**o** Si, spesso (poche volte al mese)
**o** Si, molto frequentemente(settimanalmente)
**o** Non so

**2. Hai mai avuto la sensazione che, intorno a te, tutto non fosse reale, come se non stesse realmente accadendo?**

**o** Mai
**o** Si, molto raramente (meno di 1 volta l' anno)
**o** Si, qualche volta (poche volte l'anno)
**o** Si, spesso (poche volte al mese)
**o** Si, molto frequentemente(settimanalmente)
**o** Non so

**3. Nota:** *la domanda seguente riguarda l'opposto della sensazione di "riconoscimento"!*
**Hai mai avuto la sensazione di NON aver vissuto qualcosa prima, sebbene in realtà l'avessi già vissuta?**
**Per esempio: Vedi qualcosa e/o qualcuno che conosci molto bene, ma hai la sensazione di non averlo mai visto prima!**

**o** Mai
**o** Si, molto raramente (meno di 1 volta l' anno)
**o** Si, qualche volta (poche volte l'anno)
**o** Si, spesso (poche volte al mese)
**o** Si, molto frequentemente(settimanalmente)
**o** Non so

**4. Ti è mai capitato di aver vissuto qualcosa che si era verificato prima in un sogno?**

**o** Mai
**o** Si, molto raramente (meno di 1 volta l' anno)
**o** Si, qualche volta (poche volte l'anno)
**o** Si, spesso (poche volte al mese)
**o** Si, molto frequentemente(settimanalmente)
**o** Non so

**5. Hai mai avuto la sensazione che qualcosa che ti stava accadendo non stesse capitando a te, ma a qualcun altro, come se stessi guardando te stesso dall'esterno?**

o Mai
o Molto raramente (meno di 1 volta l' anno)
o Qualche volta (poche volte l'anno)
o Spesso (poche volte al mese)
o Molto frequentemente(settimanalmente)
o Non so

**6 . Credi di essere una persona con doti paranormali?**
*(Con "Doti paranormali" si intende telepatia, chiaroveggenza, abilità psichiche, ecc.)*

o No
o No, ma non sono sicuro
o Si, ma non sono sicuro
o Si
o Non so

**7. Quante volte riesci a ricordare un sogno talmente bene da poterlo raccontare a qualcuno?**

**o** Mai
**o** Si, molto raramente (meno di 1 volta l' anno)
**o** Si, qualche volta (poche volte l'anno)
**o** Si, spesso (poche volte al mese)
**o** Si, molto frequentemente(settimanalmente)
**o** Non so

**8. Quante volte l'anno viaggi percorrendo almeno 100 km dalla zona in cui vivi ?**

**o** Mai
**o** Si, molto raramente (meno di 1 volta l' anno)
**o** Si, qualche volta (poche volte l'anno)
**o** Si, spesso (poche volte al mese)
**o** Si, molto frequentemente(settimanalmente)
**o** Non so

**9. Ti è mai capitato di sognare ad occhi aperti?**

**o** Mai
**o** Si, molto raramente (meno di 1 volta l' anno)
**o** Si, qualche volta (poche volte l'anno)
**o** Si, spesso (poche volte al mese)
**o** Si, molto frequentemente(settimanalmente)
**o** Non so

Se hai risposto "**Mai**" o "**Non so**" alla prima domanda di pagina 1, non è necessario proseguire con il questionario.

Per favore, controlla se hai risposto a tutte le domande.

Ti ringraziamo moltissimo per la tua preziosa collaborazione !

Se invece hai risposto "Si,.." alla prima domanda di pagina 1, vai alla pagina successiva.

**INTRODUZIONE**

**Rispondi alle seguenti domande solo se hai risposto "Si,…" alla prima domanda di pagina 1.**

Queste domande riguardano la tua sensazione di "*riconoscimento*".

Per *"Riconoscimento"* si intende la sensazione di aver sperimentato qualcosa prima esattamente allo stessa maniera sebbene in realtà stesse capitando per la prima volta.

**1.** Una persona può avere una sensazione di 'riconoscimento' in molti modi diversi. Può avere a che fare con uno specifico luogo, una situazione, un'attività, un avvenimento, l'incontro con qualcuno, una conversazione, un pensiero, la lettura di un libro o di un giornale …

**Hai mai avuto questa sensazione di 'riconoscimento' in uno o più dei seguenti casi?**

**(Nota: Puoi rispondere 'Sì' a più di un'opzione, per questa domanda. Ti preghiamo di rispondere a tutte le opzioni, anche se la risposta è 'No'. Per ogni opzione di cui non sei sicuro, rispondi "No")**

| | | |
|---|---|---|
| a. Un determinato | 0 Si | 0 No |
| posto……………………………………………… | 0 Si | 0 No |
| b. Una determinata situazione…………………………………………… | 0 Si | 0 No |
| c. Svolgendo una determinata attività………………………………… | 0 Si | 0 No |
| d. Un determinato episodio……………………………………………... | 0 Si | 0 No |
| e. Incontrando qualcuno……………………………………………… | 0 Si | 0 No |
| f. Raccontando a qualcuno qualcosa…………………………….. | 0 Si | 0 No |
| g. Ascoltando una conversazione, musica o una dichiarazione….. | 0 Si | 0 No |
| h. Mentre hai un determinato pensiero…………………………….... | 0 Si | 0 No |
| i. Leggendo qualcosa…………………………………………….... | 0 Si | 0 No |
| **j.** In altre situazioni diverse da quelle riportate sopra (a-i) …………... | | |

*Continua……*

**2. Mentre provi questa sensazione di “riconoscimento,” riesci a ricordare esattamente <u>dove e quando</u> hai avuto la stessa esperienza?**

o No
o Lo ricordo vagamente
o Si, lo ricordo esattamente
o Non so

**3. Quando si è verificata per l’ultima volta questa sensazione di “riconoscimento”?**

o Più di 5 anni fa
o Da 1 a 5 anni fa
o Da 6 mesi a 1 anno fa
o Da 2 a 6 mesi fa
o Da 1 a 2 mesi fa
o Nell’ultimo mese
o Non so

**4. Quanto dura in genere questa sensazione di “riconoscimento”?**

o 1 secondo o meno
o Pochi secondi
o 1 o pochi minuti
o Da 30 minuti a 1 ora
o Poche ore
o Più di qualche ora
o Non so

**5. Di solito, questa sensazione di “riconoscimento”, si riferisce a una parte di un’esperienza/situazione o la comprende del tutto?**

o Tutto
o Una parte
o Dipende
o Non so

**6. Questa sensazione di “riconoscimento” si verifica di solito in uno specifico momento della giornata?**

o No
o Al mattino, appena sveglio
o Di giorno

o Subito prima o subito dopo essere andato a letto
o Non so

**7. Mentre provi questa sensazione di "riconoscimento", ti viene mai in mente di poter prevedere quel che accadrà nei minuti immediatamente successivi?**

o Mai
o Molto raramente (meno di 1 volta l' anno)
o Qualche volta (poche volte l'anno)
o Spesso (poche volte al mese)
o Molto frequentemente(settimanalmente)
o Non so

8. **Mentre provi questa sensazione di "riconoscimento", ti sei mai sentito come se non stesse capitando a te ma piuttosto a qualcun altro, come se tu stessi guardando te stesso dall'esterno?**

o No
o Ho la vaga sensazione che non stia capitando a me
o Ho la netta sensazione che non stia capitando a me
o Ho la vaga sensazione di guardare me stesso dell'esterno
o Ho la netta sensazione di guardare me stesso dall'esterno
o Non lo so

**9. Solitamente, questa sensazione di "riconoscimento" consiste in una esatta ripetizione del passato o si riferisce piuttosto alla stessa circostanza in modo approssimativo?**

o Esattamente la stessa cosa
o Quasi esattamente la stessa cosa
o La stessa cosa
o Approssimativamente la stessa cosa
o Vagamente la stessa cosa
o Non so

**10. Contemporaneamente a questa sensazione di riconoscimento, ti sei mai sentito come se tutto intorno a te non fosse reale, come se non stesse accadendo davvero?**

o Mai
o Si, un po' irreale
o Si, vagamente irreale
o Si, irreale
o Si, totalmente irreale
o Non lo so

*Continua……*

## INTRODUZIONE

**Puoi rispondere "Si" a più di una delle opzioni delle seguenti domande**

Se *non sei sicuro*, rispondi "No".

Ti preghiamo di rispondere a tutte le opzioni, anche se la risposta è 'No'.

---

**11. In generale, quanto questa sensazione di "riconoscimento" influenza la tua vita?**

| | | |
|---|---|---|
| a. Mi lascia indifferente…………………………………………………… | 0 Si | 0 No |
| b. Mi spaventa…………………………………………………………… | 0 Si | 0 No |
| c. E' rassicurante………………………………………………………… | 0 Si | 0 No |
| d. E' bella e piacevole……………………………………………….... | 0 Si | 0 No |
| e. E'spiacevole o oppressiva…………………………………………… | 0 Si | 0 No |
| f. E' sorprendente, affascinante………………………………………... | 0 Si | 0 No |
| g. Interrompe qualsiasi cosa stia facendo……………………………….. | 0 Si | 0 No |
| **h.** Altri effetti: | | |

**12. Quale credi sia la spiegazione di questa sensazione di "riconoscimento"?**

| | | |
|---|---|---|
| **a.** Ansia o tensione | 0 Si | 0 No |
| ……………………………………………………………….. | 0 Si | 0 No |
| **b.** Scarsa memoria………………………………………………………… | 0 Si | 0 No |
| **c.** Ricordi | 0 Si | 0 No |
| inconsapevoli……………………………………………………….. | 0 Si | 0 No |
| **d.** Reincarnazione……………………………………………..................... | 0 Si | 0 No |
| **e.** Problemi di concentrazione………………………………………………… | 0 Si | 0 No |
| **f.** Doti paranormali | | |

**g.** Desiderio di fuggire dalla realtà ……………………………………………

**h.** Altre spiegazioni:

**13. Come ti senti generalmente prima di avvertire questa sensazione di 'riconoscimento'?**

| | | |
|---|---|---|
| a. Mentalmente affaticato..……………………………………………… | 0 Si | 0 No |
| b. Malinconico o depresso..…………………………………………… | 0 Si | 0 No |
| c. Nervoso o teso……..……………………………………………… | 0 Si | 0 No |
| d. Fisicamente affaticato..………………………………..................... | 0 Si | 0 No |
| e. Allegro e felice…………………………..…………………………. | 0 Si | 0 No |
| f. Confuso o con la testa vuota ……………..……………………….. | 0 Si | 0 No |
| g. Rilassato……………………………….......................................... | 0 Si | 0 No |
| h. Arrabbiato..…………………………………………….................. | 0 Si | 0 No |
| . | 0 Si | 0 No |
| i. Spaventato…………………………………………………………. | 0 Si | 0 No |
| j. Assonnato………………………………………………………… | 0 Si | 0 No |

**k.** Fisicamente malato……………………………………………………

**14. Hai mai provato questa sensazione di "riconoscimento"durante una delle seguenti condizioni?**

| | | |
|---|---|---|
| **a.** Mal di testa ……………………………………………… | 0 Si | 0 No |
| ………… | 0 Si | 0 No |
| **b.** Momenti di "Black out"……………………………………………… | 0 Si | 0 No |
| **c.** Crisi epilettiche…………………………………………………….. | 0 Si | 0 No |
| **d.** Piena concentrazione | 0 Si | 0 No |

**e.** Assunzione di alcolici…………………..………………………………...

**La domanda n° 14 era l'ultima. Per piacere, controlla se hai risposto a tutte le domande. Grazie della collaborazione!**
